# Poultry Consumption and Arsenic Exposure in the U.S. Population

**DOI:** 10.1289/EHP351

**Published:** 2016-10-13

**Authors:** Anne E. Nigra, Keeve E. Nachman, David C. Love, Maria Grau-Perez, Ana Navas-Acien

**Affiliations:** 1 Department of Environmental Health Sciences, Columbia University Mailman School of Public Health, New York, New York, USA; 2 Department of Epidemiology,; 3 Department of Environmental Health Sciences,; 4 Center for a Livable Future, and; 5 Department of Health Policy and Management, Johns Hopkins Bloomberg School of Public Health, Baltimore, Maryland, USA

## Abstract

**Background:**

Arsenicals (roxarsone and nitarsone) used in poultry production likely increase inorganic arsenic (iAs), monomethylarsonic acid (MMA), dimethylarsinic acid (DMA), and roxarsone or nitarsone concentrations in poultry meat. However, the association between poultry intake and exposure to these arsenic species, as reflected in elevated urinary arsenic concentrations, is unknown.

**Objectives:**

Our aim was to evaluate the association between 24-hr dietary recall of poultry consumption and arsenic exposure in the U.S. population. We hypothesized first, that poultry intake would be associated with higher urine arsenic concentrations and second, that the association between turkey intake and increased urine arsenic concentrations would be modified by season, reflecting seasonal use of nitarsone.

**Methods:**

We evaluated 3,329 participants ≥ 6 years old from the 2003–2010 National Health and Nutrition Examination Survey (NHANES) with urine arsenic available and undetectable urine arsenobetaine levels. Geometric mean ratios (GMR) of urine total arsenic and DMA were compared across increasing levels of poultry intake.

**Results:**

After adjustment, participants in the highest quartile of poultry consumption had urine total arsenic 1.12 (95% CI: 1.04, 1.22) and DMA 1.13 (95% CI: 1.06, 1.20) times higher than nonconsumers. During the fall/winter, participants in the highest quartile of turkey intake had urine total arsenic and DMA 1.17 (95% CI: 0.99, 1.39; *p*-trend = 0.02) and 1.13 (95% CI: 0.99, 1.30; *p*-trend = 0.03) times higher, respectively, than nonconsumers. Consumption of turkey during the past 24 hr was not associated with total arsenic or DMA during the spring/summer.

**Conclusions:**

Poultry intake was associated with increased urine total arsenic and DMA in NHANES 2003–2010, reflecting arsenic exposure. Seasonally stratified analyses by poultry type provide strong suggestive evidence that the historical use of arsenic-based poultry drugs contributed to arsenic exposure in the U.S. population.

**Citation:**

Nigra AE, Nachman KE, Love DC, Grau-Perez M, Navas-Acien A. 2017. Poultry consumption and arsenic exposure in the U.S. population. Environ Health Perspect 125:370–377; http://dx.doi.org/10.1289/EHP351

## Introduction

In populations with low arsenic levels in drinking water, exposure to inorganic arsenic (iAs) occurs mainly through diet, particularly through the consumption of rice and other grains, as well as some juices and wine [Davis et al. 2012; deCastro et al. 2014; U.S. Food and Drug Administration (FDA) 2014a; Navas-Acien et al. 2011]. iAs is a toxic and carcinogenic metalloid that occurs naturally in water, air, and soil and enters the food supply through geological releases, contaminated water, and anthropogenic sources such as pesticide residue, nonferrous metal smelting, and waste incineration [Agency for Toxic Substances and Disease Registry (ATSDR) 2007]. Increasing evidence suggests that mono- and dimethylated metabolites of inorganic arsenic [monomethylarsonate (MMA) and dimethylarsinate (DMA), respectively] may cause oxidative stress and cytotoxicity and may be carcinogenic depending on the valence state [Hughes 2002; International Agency for Research on Cancer (IARC) 2012a]. Little is known, however, about the potential independent contribution of poultry intake to arsenic exposure in human populations.

Arsenic-based drugs (roxarsone, used in chickens, and nitarsone, used in turkeys) were deliberately used in U.S. poultry production for decades, potentially representing an unnecessary and easily controllable source of exposure to iAs, MMA, DMA, and roxarsone/nitarsone in the population (Liu et al. 2016; Nachman et al. 2016; Silbergeld and Nachman 2008). The FDA withdrew marketing approvals for roxarsone and for two other arsenic-based feed additives in 2013, and they withdrew approval for nitarsone, which is used to prevent histomoniasis in turkeys, in December of 2015 (FDA 2014b, 2015; Abraham et al. 2013). Historical use of nitarsone in turkey production and of roxarsone in chicken production may thus have been a chronic source of arsenic exposure for the U.S. population and may be ongoing in other parts of the world (Yao et al. 2013).

In 2010, it was estimated that ~88% of broiler chickens available at market had been treated with roxarsone (Nachman et al. 2012). A similar estimate is not available for nitarsone, but turkey industry representatives have reported that nitarsone was used seasonally during hot-weather months in young turkeys that were consumed during the fall/winter (Aubrey 2013). Analyses of chicken meat have shown that the use of roxarsone during chicken production likely contributes to elevated iAs, DMA, and other unknown arsenic species in chicken meat and that the concentration of iAs increases with cooking (Nachman et al. 2013). Analyses of turkey meat have also shown that the use of nitarsone during turkey production likely contributes to elevated iAs, MMA, and other unknown arsenic species in turkey meat (Nachman et al. 2016). In a recent feeding study, iAs, MMA, DMA, roxarsone, and an unidentified roxarsone metabolite were elevated in the meat of chickens fed a roxarsone-supplemented diet compared with the levels of those compounds in chickens fed a non-roxarsone diet (Liu et al. 2016). However, it is unknown if consumption of poultry exposed to arsenic-based drugs results in increased arsenic exposure and internal dose in the population, as reflected in urinary excretion.

The National Health and Nutrition Examination Survey (NHANES) collects 24-hr dietary recall information the same day that a spot urine sample is collected for total and speciated arsenic analysis [U.S. National Center for Health Statistics (NCHS) 2014]. Previous studies in NHANES have evaluated poultry consumption as a potential confounder of dietary arsenic exposure (Davis et al. 2012) or in analyses without accounting for other dietary sources of arsenic such as seafood (deCastro et al. 2014). Our objective was to evaluate whether consumption of poultry in the past 24 hr was associated with increased arsenic exposure as measured in urine by total arsenic and DMA during a time period when arsenic-based poultry drugs were approved for use in the United States.

First, we hypothesized that poultry intake would be associated with higher arsenic exposure as determined in urine. Second, we hypothesized that the association between turkey intake and elevated urine arsenic would be modified by season (strongest for turkey consumed during the fall/winter and null for turkey consumed during the spring/summer), whereas the association for chicken intake would persist across seasons. To our knowledge, this is the first study to evaluate the independent association between recent poultry consumption and arsenic exposure as reflected in urine total arsenic and DMA concentrations.

## Methods

### Study Population

We analyzed data from the 2003–2010 cycles of the NHANES, conducted by the NCHS. NHANES is a multi-stage, nationally representative sample of the noninstitutionalized population (NCHS 2014). Our study used data from the demographic questionnaire, the 24-hr dietary recall, the clinical examination, and the laboratory examination. All NHANES protocols were approved by the NCHS institutional review board (IRB), and all participants gave written informed consent (NCHS 2011a). Our study was exempt from IRB approval because we used de-identified, publicly available data. To capture a time period when urine arsenic measures were available while roxarsone, nitarsone, and other arsenic-based drugs were still available in poultry production, we restricted our analysis to 2003–2010 NHANES cycles.

Urine arsenic was measured in a one-third subsample of all participants ≥ 6 years of age. From 10,451 participants in the NHANES 2003–2010 urine arsenic subsamples, we excluded 387 missing total urine arsenic, arsenobetaine, or DMA; 229 who were pregnant; 880 who were missing BMI, cotinine, urinary creatinine, or education information; and 5,626 with detectable arsenobetaine because seafood arsenicals markedly contribute to total arsenic exposure and DMA and make it difficult to evaluate the contribution of other foods to iAs exposure (Navas-Acien et al. 2011). The final sample included 3,329 participants who were ≥ 6 years old. The response rate across the entire survey period was 76.5% (NCHS 2013).

### Urine Arsenic

Spot urine samples were collected during examination, poured in 5-mL cryovial vessels, frozen at ≤ –20°C, and shipped within 1 week on dry ice to the National Center for Environmental Health (NCEH) at the Centers for Disease Control and Prevention for analysis (NCHS 2005). Total arsenic concentrations were determined via quadrupole inductively coupled plasma–mass spectrometry with dynamic reaction cell (ICP-DRC-MS). Speciated arsenic concentrations (arsenite, arsenate, MMA, DMA, and arsenobetaine) were determined via high-performance liquid chromatography (HPLC) coupled to ICP-DRC-MS (NCHS 2007, 2009a, 2011b, 2011c).

We used total arsenic and DMA concentrations in urine, but not arsenite, arsenate, or MMA as the limits of detection (LOD); percents of analytic sample below the LOD for these species [arsenite (1.2 μg/L, 97.7%), arsenate (1.0 μg/L, 96.8%), and MMA (0.9 μg/L, 74.0%)] were high compared to those in other studies evaluating urinary arsenic levels, and in most samples, these species were largely undetectable (Cubadda et al. 2012; NCHS 2007, 2009a, 2011b, 2011c; Scheer et al. 2012). Neither nitarsone nor roxarsone was measured in urine. For arsenobetaine, the LOD was 0.4 μg/L, and 37% of the participants had undetectable concentrations. Detectable arsenobetaine in NHANES has shown to be an excellent biomarker of recent seafood intake (Navas-Acien et al. 2011). Restricting our study population to participants with undetectable arsenobetaine likely removed the contribution of seafood arsenicals to both total arsenic and DMA, which could then be interpreted as biomarkers of arsenic exposure not derived from seafood (Liu et al. 2016; Navas-Acien et al. 2011).

The LOD for total arsenic ranged from 0.60 to 0.74 μg/L across the entire survey period, with an inter-assay coefficient of variation ranging from 3.0% to 19.4% for lots with mean concentrations of 3.6 to 8.15 μg/L (NCEH 2006a, 2007, 2008, 2010). For DMA across the entire survey period, the LOD was 1.7 μg/L with an inter-assay coefficient of variation ranging from 4.6% to 6.6% for lots with mean concentrations of 4.12 to 6.85 μg/L (NCEH 2004, 2006b, 2008, 2010). The percents of participants in the analytic sample below the LOD for poultry consumers and nonconsumers were 1.2% and 4.0%, respectively, for urine total arsenic and 28.2% and 32.0%, respectively, for DMA. Values below the LOD for total arsenic and DMA were replaced by the LOD divided by the square root of 2. The LOD for arsenobetaine was < 0.4 μg/L across the entire survey period (NCEH 2004, 2006b, 2008, 2010).

### 24-Hr Poultry Intake Assessment

Poultry intake during the past 24 hr was collected via multiple-pass dietary recall during the in-person questionnaire. Multiple-pass dietary recall is the validated method of choice for food recall and is conducted in five steps: *a*) easily remembered foods; *b*) frequently forgotten foods; *c*) time and occasion of meals; *d*) detailed descriptions, eating locations, and portions; and *e*) final review probe (Conway et al. 2004; NCHS 2009b). To estimate portion size, participants are given 2- and 3-dimensional measuring guides (NCHS 2010). Food and drink items are reported in grams of intake and linked to eight-digit U.S. Department of Agriculture (USDA) food codes. Because USDA food codes often contain multiple food components (e.g., “chicken sandwich”), we used Food Commodity Index Database (FCID) codes to determine the weight of each USDA food item attributable to poultry meat (see Table S1) (Joint Institute for Food Safety and Applied Nutrition 2010). The FCID was developed by the U.S. Environmental Protection Agency’s (EPA’s) Office of Pesticide Programs. FCID codes convert the weight of each USDA food item into the respective weights of all commodities included in the item. For each participant, FCID commodity codes are summed across all USDA food items. We analyzed FCID commodity codes for “Turkey, meat” and “Chicken, meat” and defined poultry intake as the sum of chicken and turkey meat intake, in grams per kilogram body weight per day as recommended by the U.S. EPA and by the Food and Agriculture Organization [U.S. EPA 2011; World Health Organization (WHO) 2005]. However, some USDA food code items are not specific and are listed as containing either chicken or turkey (e.g., “‘chicken or turkey’ soup”). Participants may thus be assigned as having chicken and turkey in the past 24 hr if they ate both commodities or if the food item assigned contained both chicken and turkey in the description.

To control for potential confounding by other foods that may have contained substantial amounts of arsenic, we used FCID commodity codes corresponding to rice, wine, and juice intake (see Table S1) (FDA 2014a). Because no FCID commodity codes exist for cereals, we used corresponding USDA food codes (see Table S2).

### Other Variables

Questionnaire data (age, sex, race/ethnicity, education, smoking status, poverty–income ratio), examination data (body mass index, urine creatinine, serum cotinine), and tap water source were also available from NHANES. We categorized race/ethnicity as non-Hispanic white/non-Hispanic black/Mexican-American/other, including multiple races. Smoking status in adults was defined as never/former/current by self-report. Children (< 20 years old) who never smoked a whole cigarette were categorized as “never” smokers; children who smoked a whole cigarette but not in the past 30 days were categorized as “former” smokers; children who smoked a cigarette in the last 30 days were categorized as “current” smokers. All participants with serum cotinine ≥ 10 ng/mL were recategorized as “current” smokers, and children missing self-reported smoking status with serum cotinine < 10 ng/mL were categorized as “never” smokers.

### Statistical Analysis

All statistical analyses were performed using the ‘survey’ package in R (version 3.30; R Project for Statistical Computing) to account for NHANES complex survey design and sampling weights (Lumley 2014). Both urine total arsenic and DMA were right-skewed and were natural log–transformed for analysis.

We compared the geometric mean ratios (GMRs) and corresponding 95% confidence intervals (CIs) for both total arsenic and DMA by poultry intake using multiple linear regression across categories of intake for poultry and for chicken and turkey separately, comparing quartiles of intake within those who reported consuming poultry, chicken, or turkey with a reference category that included those who did not report any poultry, chicken, or turkey intake, respectively. Model 1 adjusted for urine creatinine (natural log–transformed continuous), age (continuous), sex (male/female), race/ethnicity (non-Hispanic white/non-Hispanic black/Mexican-American/other, including multiple), education (less than high school/high school or equivalent/greater than high school), poverty–income ratio (continuous), body mass index (continuous), smoking status (never/former/current), serum cotinine (natural log–transformed continuous), and tap water source (community supply/well or cistern/spring/other/no tap water). We were unable to exclude participants living in areas with high iAs levels in drinking water because no information about participant location or geography was publicly available. Model 2 further adjusted for past-24-hr intake of rice, cereal, juice, and wine (grams per kilogram body weight, continuous). To allow a more flexible dose–response analysis, we also analyzed poultry intake as natural log–transformed continuous with restricted quadratic splines at the 10th, 50th, and 90th percentiles of poultry consumption among consumers, defining those who reported no poultry consumption as the reference group.

We also conducted subgroup analyses for poultry consumption by age, sex, race/ethnicity, and rice consumption using multiple linear regression on natural log–transformed total arsenic and DMA, with interaction terms for each subgroup. We then estimated the GMR of total arsenic and DMA, comparing the 75th percentile with the 25th percentile of the poultry intake distribution, including nonconsumers, by subgroup. To determine if season modified the relationship between intake and urine arsenic for turkey but not chicken, we stratified our analyses by fall/winter (1 November–30 April) and spring/summer (1 May–31 October). We hypothesized that for turkey meat, but not chicken meat, the association would attenuate in the spring/summer but remain positive in the fall/winter, reflecting the seasonal use of nitarsone in turkey production and the yearlong use of roxarsone in chicken production. Specifically, we hypothesized that summer use of nitarsone in turkey production would result in arsenic exposure as reflected in elevated urine total arsenic and DMA levels in consumers during the fall/winter only. As a sensitivity analysis, we repeated the main analysis for DMA levels by quartile of intake and poultry type both overall and stratified by season, imputing DMA levels below the LOD using multiple imputation chained equations via the “mice” package in R (R Project for Statistical Computing), with total urine arsenic, tap water source, and juice, wine, and rice intake as predictors of undetectable DMA levels instead of using the default replacement (LOD divided by the square root of 2).

## Results

### Participant Characteristics by Poultry Consumption

The weighted prevalence (national estimate) of poultry intake in the past 24 hr was 52% overall and 50% among those with undetectable arsenobetaine (Table 1). Poultry consumers were younger, more likely to belong to racial/ethnic minority groups, more likely to report consuming rice and juice in the past 24 hr, and less likely to report consuming cereals in the past 24 hr. Among those with undetectable arsenobetaine, the median concentrations of urine total arsenic and DMA were 4.18 and 2.56 μg/L, respectively, among poultry consumers, and 3.99 and 2.42 μg/L, respectively, among nonconsumers.

**Table 1:** Participant characteristics by arsenobetaine and poultry intake, 2003–2010.

Characteristic	Participants with undetectable arsenobetaine (*n* = 3,329) Poultry intake past 24 hr		All participants (*n* = 8,955) Poultry intake past 24 hr	
	Yes	No	Yes	No
*n* (%)^*a*^	1,740 (50.1)	1,589 (49.9)	4,773 (52.0)	4,182 (48.0)
Age, years [mean (SE)]	35.2 (0.6)	38.2 (0.8)	39.3 (0.4)	41.8 (0.5)
Sex, % female (SE)	53.9 (1.5)	53.8 (1.7)	50.4 (1.1)	50.6 (1.0)
Race/ethnicity, % (SE)
Non-Hispanic white	70.0 (2.2)	76.2 (2.0)	65.4 (2.0)	73.7 (1.8)
Non-Hispanic black	10.4 (1.1)	7.5 (0.9)	13.6 (1.1)	9.2 (0.7)
Mexican-American	11.6 (1.5)	9.0 (1.0)	9.7 (1.1)	8.7 (0.9)
Other, including multiple	7.9 (0.9)	7.3 (1.0)	11.3 (1.0)	8.4 (0.8)
Education, % (SE)
< High school	22.0 (1.5)	20.0 (1.4)	19.0 (1.0)	18.7 (1.0)
High school or equivalent	26.7 (1.6)	28.8 (1.7)	23.8 (0.8)	26.9 (0.9)
> High school	51.4 (2.0)	51.2 (1.9)	54.4 (1.2)	57.2 (1.2)
Smoking, % (SE)
Never	58.3 (1.9)	59.0 (1.9)	57.5 (1.2)	52.8 (1.1)
Former	15.9 (1.1)	15.5 (1.4)	19.1 (0.8)	19.9 (0.9)
Current	25.8 (1.9)	25.4 (1.7)	23.4 (0.9)	27.3 (1.0)
Cotinine, nmol/L^*b*^	0.07 (0.02–6.80)	0.08 (0.02–3.78)	0.06 (0.02–2.17)	0.07 (0.02–13.80)
Body mass index, kg/m^2^ [mean (SE)]	26.2 (0.2)	26.3 (0.2)	27.1 (0.1)	27.2 (0.2)
Chicken past 24-hr, *n* (%)^*c*^	1,622 (93.5)	—	4,459 (93.7)	—
Turkey past 24-hr, *n* (%)	752 (46.3)	—	2,093 (47.0)	—
Both past 24-hr, *n* (%)	634 (39.8)	—	1,779 (40.7)	—
Seafood past 24-hr, % (SE)	3.4 (0.6)	2.2 (0.5)	16.7 (0.8)	16.8 (0.9)
Rice past 24-hr, % (SE)	18.9 (1.2)	10.7 (1.2)	26.7 (1.1)	14.9 (1.0)
Juice past 24-hr, % (SE)	19.8 (1.2)	16.3 (1.4)	17.8 (0.7)	15.2 (0.7)
Wine past 24-hr, % (SE)	3.2 (0.7)	3.6 (0.6)	6.4 (0.6)	6.1 (0.6)
Cereal past 24-hr, % (SE)	29.2 (1.7)	33.0 (2.2)	29.7 (0.9)	31.5 (1.2)
Total urine arsenic, μg/L	4.18 (2.47–6.97)	3.99 (2.21–6.30)	8.22 (4.35–16.49)	7.63 (4.00–16.17)
Urine DMA, μg/L	2.56 (1.20–4.26)	2.42 (1.20–3.93)	3.73 (2.11–6.00)	3.52 (2.00–5.93)
Urine AB (μg/L)	0.28 (0.28–0.28)	0.28 (0.28–0.28)	1.20 (0.28–5.89)	0.94 (0.28–5.38)
Total arsenic minus AB (μg/L)	3.90 (2.19–6.69)	3.70 (1.93–6.00)	6.10 (3.34–10.70)	5.77 (3.12–10.47)
Notes: AB, arsenobetaine; DMA, dimethylarsinate; SE, standard error of the mean. ^***a***^All percentages are weighted to account for NHANES complex sampling design and weights. ^***b***^Cotinine, total urine arsenic, DMA, AB, and total arsenic minus AB are described with median (interquartile range). ^***c***^Consumers for food items (poultry, seafood, rice, juice, wine, cereal) are identified by reported consumption of any USDA food code item containing that commodity in the 24-hr recall.				

### Urine Arsenic by Poultry Intake

**Figure 1:**
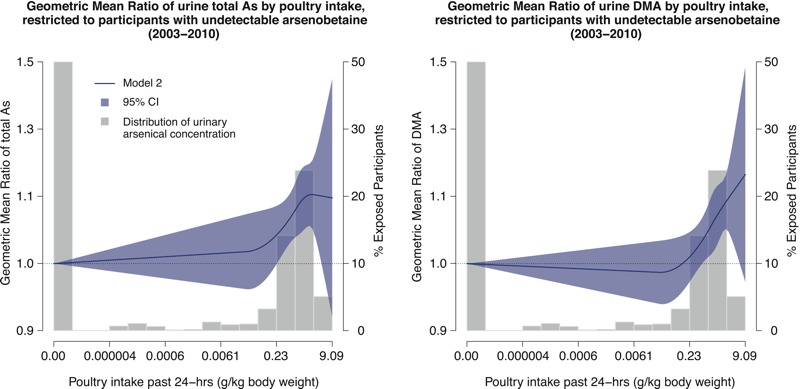
Geometric mean ratio (95% confidence interval) of urine total arsenic (As) and dimethylarsinate (DMA) by poultry intake in the past 24 hr. Lines represent the geometric mean ratio of urinary arsenical concentrations by levels of poultry intake (grams/kilogram body weight/day), based on restricted quadratic spline models with knots at the 10th, 50th, and 90th percentiles of natural log–transformed poultry intake (left *y*-axis). Blue shaded areas surrounding the lines represent 95% confidence intervals. Shaded gray bars represent the distribution of poultry intake (grams/kilogram body weight) within the study population and are shown as “percent exposed participants” in the right *y*-axis. Geometric mean ratios were adjusted for urinary creatinine (natural log–transformed continuous), age (continuous), sex (male/female), race/ethnicity (non-Hispanic white/non-Hispanic black/Mexican-American/other, including multiple), education (< high school/high school or equivalent/> high school), body mass index (continuous), smoking status (never/former/current), serum cotinine (natural log–transformed continuous), poverty income ratio (PIR, continuous), tap water source (community supply/well, cistern/spring/other/no tap water), and past 24 hr intake of rice, cereal, juice, and wine (grams/kilogram body weight, continuous). Poultry, rice, juice, and wine intake were derived from Food Commodity Index Database (FCID) codes and analyzed in grams/kilogram body weight/day. Cereal intake was derived from U.S. Department of Agriculture food codes, because no FCID code exists for cereal, and were analyzed in grams/kilogram body weight/day.

**Figure 2:**
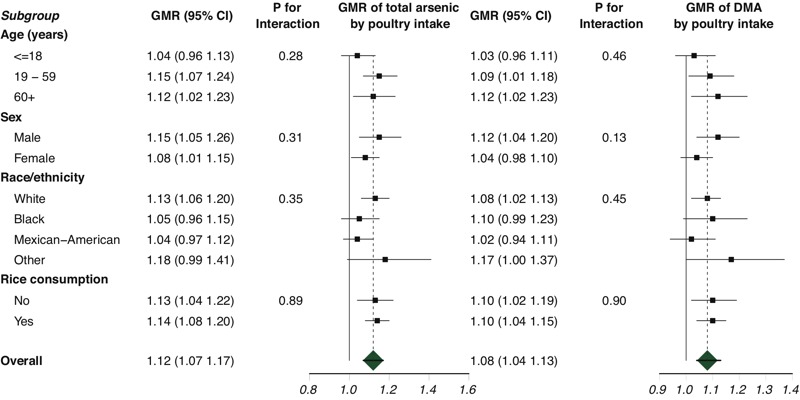
Geometric mean ratio (GMR) [95% confidence interval (CI)] of urine total arsenic and dimethylarsinate (DMA) concentrations comparing an interquartile range of poultry intake (75th to 25th percentile of poultry intake, grams/kilogram body weight) by participant subgroups. Models were adjusted for urinary creatinine (natural log–transformed continuous), age (continuous), sex (male/female), race/ethnicity (non-Hispanic white/non-Hispanic black/Mexican-American/other, including multiple), education (< high school/high school or equivalent/> high school), body mass index (continuous), smoking status (never/former/current), serum cotinine (natural log–transformed continuous), poverty income ratio (PIR, continuous), tap water source (community supply/well, cistern/spring/other/no tap water), and past 24-hr intake of rice, cereal, juice, and wine (grams/kilogram body weight, continuous). Poultry, rice, juice, and wine intake were derived from Food Commodity Index Database (FCID) codes and analyzed in grams/kilogram body weight/day. Cereal intake was derived from U.S. Department of Agriculture food codes, becuase no FCID code exists for cereal, and were analyzed in grams/kilogram body weight/day. For each subgroup analysis, the variable of interest was replaced by the subgroup indicator (e.g., age, modeled in three categories) and the interaction of the subgroups with poultry intake (continuous, grams/kilogram body weight).

After full adjustment, the GMRs (95% CIs) comparing the highest quartile of poultry intake among consumers (> 1.61 g/kg body weight per day) with nonconsumers were 1.12 (95% CI: 1.04, 1.22) for total arsenic and 1.13 (95% CI: 1.06, 1.20) for DMA (Table 2). When stratified by the type of poultry, the corresponding GMRs for total arsenic and DMA were 1.15 (95% CI: 1.06, 1.25) and 1.13 (95% CI: 1.06, 1.21) for chicken and 1.09 (95% CI: 0.99, 1.20) and 1.10 (95% CI: 1.01, 1.20) for turkey. In restricted quadratic spline models, both total arsenic and DMA increased significantly with increasing poultry intake beyond approximately 1.0 g/kg body weight (Figure 1). We found no significant difference in GMRs of total arsenic or DMA by poultry intake across age, sex, race/ethnicity, and rice consumption subgroups, although the association among children and adolescents ≤ 18 years of age appeared weaker than in adults (Figure 2). For the analyses based on imputed undetectable DMA, fully adjusted GMRs of DMA concentrations comparing participants in the highest quartile of chicken and turkey intake with non-consumers were 1.06 (95% CI: 0.99, 1.13) and 1.07 (95% CI: 0.98, 1.16), respectively.

**Table 2:** Urine arsenic concentrations by poultry intake in the past 24 hrs (*n* = 3,329).

Meat intake (g/kg body weight)	*n* (%)	Total arsenic			DMA		
		Geometric mean (95% CI)	Model 1^*a*^ GMR (95% CI)	Model 2^*b*^ GMR (95% CI)	Geometric mean (95% CI)	Model 1^*a*^ GMR (95% CI)	Model 2^*b*^ GMR (95% CI)
Poultry^*c*^
0	1,589 (49.9)^*d*^	3.72 (3.45, 4.01)	1 (reference)	1 (reference)	2.53 (2.40, 2.67)	1 (reference)	1 (reference)
0.001–0.42 (0.14)	435 (12.6)	3.63 (3.29, 4.01)	1.06 (0.99, 1.14)	1.05 (0.99, 1.13)	2.39 (2.24, 2.55)	1.02 (0.95, 1.08)	1.01 (0.95, 1.07)
0.42–0.90 (0.66)	435 (12.3)	4.11 (3.64, 4.64)	1.13 (1.04, 1.23)	1.12 (1.04, 1.22)	2.64 (2.39, 2.93)	1.08 (1.00, 1.16)	1.07 (0.99, 1.16)
0.90–1.61 (1.20)	435 (13.0)	4.12 (3.84, 4.43)	1.12 (1.04, 1.21)	1.12 (1.05, 1.21)	2.65 (2.46, 2.85)	1.08 (1.01, 1.17)	1.08 (1.01, 1.16)
1.61–9.08 (2.83)	435 (12.1)	4.60 (4.16, 5.09)	1.17 (1.08, 1.28)	1.12 (1.04, 1.22)	3.03 (2.81, 3.28)	1.17 (1.10, 1.25)	1.13 (1.06, 1.20)
*p*-Trend^*e*^			< 0.001	< 0.001		< 0.001	< 0.001
Chicken^*c*^
0	1,707 (53.2)	3.74 (3.48, 4.02)	1 (reference)	1 (reference)	2.54 (2.41, 2.67)	1 (reference)	1 (reference)
0.001–0.30 (0.09)	406 (12.2)	3.44 (3.12, 3.79)	1.02 (0.94, 1.10)	1.02 (0.95, 1.09)	2.28 (2.12, 2.46)	0.99 (0.92, 1.05)	0.98 (0.93, 1.04)
0.30–0.79 (0.55)	405 (11.4)	4.16 (3.77, 4.60)	1.13 (1.04, 1.23)	1.12 (1.04, 1.20)	2.70 (2.45, 2.97)	1.09 (1.02, 1.17)	1.08 (1.01, 1.15)
0.79–1.44 (1.07)	406 (12.3)	4.24 (3.91, 4.60)	1.17 (1.08, 1.26)	1.16 (1.08, 1.25)	2.73 (2.52, 2.97)	1.12 (1.05, 1.21)	1.12 (1.04, 1.20)
1.44–8.89 (2.63)	405 (10.9)	4.72 (4.23, 5.27)	1.19 (1.09, 1.30)	1.15, (1.06, 1.25)	3.07 (2.83, 3.34)	1.17 (1.09, 1.25)	1.13 (1.06, 1.21)
*p*-Trend^*e*^			< 0.001	< 0.001		< 0.001	< 0.001
Turkey^*c*^
0	2,577 (76.8)	3.86 (3.61, 4.12)	1 (reference)	1 (reference)	2.58 (2.45, 2.71)	1 (reference)	1 (reference)
0.001–0.14 (0.07)	188 (5.6)	3.79 (3.30, 4.36)	1.06 (0.98, 1.14)	1.04 (0.95, 1.14)	2.62 (2.33, 2.94)	1.07 (0.99, 1.17)	1.06 (0.97, 1.15)
0.14–0.29 (0.21)	188 (5.2)	3.93 (3.48, 4.43)	1.04 (0.94, 1.14)	1.07 (0.97, 1.17)	2.44 (2.22, 2.68)	0.98 (0.91, 1.06)	1.01 (0.94, 1.08)
0.29–0.54 (0.39)	188 (5.8)	3.98 (3.41, 4.65)	1.06 (0.96, 1.18)	1.06 (0.96, 1.17)	2.53 (2.19, 2.92)	1.02 (0.91, 1.14)	1.01 (0.91, 1.13)
0.54–3.29 (1.15)	188 (6.5)	4.55 (3.94, 5.25)	1.12 (1.00, 1.25)	1.09 (0.99, 1.20)	3.00 (2.63, 3.42)	1.13 (1.02, 1.26)	1.10 (1.01, 1.20)
*p*-Trend^*e*^			0.04	0.04		0.07	0.08
Notes: CI, confidence interval; GMR, geometric mean ratio. Poultry defined as chicken and/or turkey. ^***a***^Model 1 adjusted for urine creatinine (natural log–transformed continuous), age (continuous), sex (male/female), race/ethnicity (non-Hispanic white/non-Hispanic black/Mexican-American/Other, including multiple), education (< high school/high school or equivalent/> high school), body mass index (continuous), smoking status (never/former/current), serum cotinine (natural log–transformed continuous), poverty income ratio (PIR, continuous), and tap water source (community supply/well, cistern/spring/other/no tap water). ^***b***^Model 2 further adjusted for past 24 hr intake of rice, cereal, juice, and wine (grams/kilogram body weight, continuous). ^***c***^Intake values for a quantile are range (mean). ^***d***^All percentages are weighted to account for NHANES complex sampling design and weights. ^***e***^*p*-Trend obtained from adding quartile of poultry intake as continuous variable to model.							

### Stratified Analysis by Season

In analyses stratified by season (winter vs. summer), the association between chicken intake and urine total arsenic and DMA remained similar for both seasons (*p*-value for interaction = 0.76 for total arsenic and 0.24 for DMA). For turkey intake, however, the interaction was statistically significant for total arsenic [*p*-value for interaction = 0.04 for total arsenic and borderline for DMA (0.07)], with strong associations for total arsenic and DMA in the fall/winter (*p* for trend = 0.02 and 0.03, respectively) but not in the spring/summer (*p* for trend = 0.70 and 0.99, respectively) (Table 3). For the analysis based on imputed undetectable DMA, fully adjusted GMRs of DMA concentrations comparing participants in the highest quartile of intake to nonconsumers in the spring/summer for chicken and turkey were 1.02 (95% CI: 0.84, 1.11) and 1.06 (95% CI: 0.96, 1.17), respectively. During the fall/winter, GMRs of imputed DMA values comparing participants in the highest quartile of intake to nonconsumers for chicken and turkey were 1.06 (95% CI: 0.94, 1.19) and 1.08 (95% CI: 1.00, 1.17), respectively.

**Table 3:** Urine arsenic concentrations by poultry intake in the past 24 hrs stratified by season (*n* = 3,329).

Intake (g/kg body weight)^*a*^	*N* (%)	Total arsenic			DMA		
		Geometric mean (95% CI)	Model 1^*b*^ GMR (95% CI)	Model 2^*c*^ GMR (95% CI)	Geometric mean (95% CI)	Model 1^*b*^ GMR (95% CI)	Model 2^*c*^GMR (95% CI)
Turkey intake
Spring/summer season
0	1,410 (77.5)^*d*^	3.71 (3.40, 4.05)	1 (reference)	1 (reference)	2.50 (2.35, 2.66)	1 (reference)	1 (reference)
0.001–0.15 (0.06)	104 (6.0)	3.65 (3.00, 4.44)	1.02 (0.90, 1.14)	1.00 (0.88, 1.14)	2.61 (2.24, 3.05)	1.10 (0.98, 1.23)	1.08 (0.96, 1.23)
0.15–0.29 (0.21)	101 (4.9)	3.69 (3.13, 4.33)	0.99 (0.84, 1.16)	1.02 (0.88, 1.17)	2.27 (1.99, 2.58)	0.93 (0.81, 1.08)	0.95 (0.84, 1.08)
0.29–0.53 (0.39)	102 (6.0)	3.64 (3.00, 4.42)	1.02 (0.89, 1.16)	1.02 (0.90, 1.16)	2.28 (1.94, 2.68)	0.93 (0.82, 1.06)	0.94 (0.82, 1.07)
0.53–3.18 (1.29)	102 (5.6)	3.94 (3.29, 4.70)	1.02 (0.91, 1.14)	1.01 (0.92, 1.12)	2.65 (2.26, 3.12)	1.07 (0.95, 1.20)	1.06 (0.96, 1.17)
*p*-Trend^*e*^			0.75	0.70		0.98	0.99
Fall/winter season
0	1,167 (75.6)	4.12 (3.80, 4.47)	1 (reference)	1 (reference)	2.74 (2.57, 2.92)	1 (reference)	1 (reference)
0.001–0.14 (0.08)	86 (5.2)	4.23 (3.60, 4.97)	1.13 (1.00, 1.27)	1.11 (1.00, 1.24)	2.68 (2.26, 3.18)	1.04 (0.92, 1.18)	1.03 (0.92, 1.15)
0.14–0.28 (0.22)	86 (5.9)	4.13 (3.57, 4.79)	1.13 (0.96, 1.32)	1.16 (1.00, 1.34)	2.65 (2.36, 2.98)	1.08 (0.97, 1.21)	1.11 (1.02, 1.22)
0.28–0.56 (0.40)	85 (5.3)	4.79 (3.82, 6.00)	1.10 (0.91, 1.32)	1.06 (0.90, 1.26)	3.07 (2.41, 3.90)	1.13 (0.93, 1.37)	1.09 (0.92, 1.30)
0.56–3.29 (1.03)	86 (7.9)	5.38 (4.33, 6.69)	1.22 (1.01, 1.48)	1.17 (0.99, 1.39)	3.47 (2.87, 4.20)	1.19 (1.00, 1.40)	1.13 (0.99, 1.30)
*p*-Trend^*e*^			0.013	0.018		0.02	0.03
*p*-Interaction^*f*^			0.030	0.044		0.05	0.07
Chicken intake
Spring/summer season
0	994 (55.8)	3.63 (3.30, 3.99)	1 (reference)	1 (reference)	2.48 (2.32, 2.64)	1 (reference)	1 (reference)
0.001–0.26 (0.09)	209 (11.4)	3.31 (2.90, 3.78)	1.02 (0.93, 1.12)	1.01 (0.94, 1.10)	2.21 (2.02, 2.42)	0.97 (0.91, 1.05)	0.97 (0.90, 1.04)
0.26–0.77 (0.52)	204 (10.8)	3.87 (3.31, 4.53)	1.10 (1.00, 1.22)	1.10 (1.00, 1.21)	2.56 (2.17, 3.02)	1.07 (0.96, 1.20)	1.07 (0.96, 1.18)
0.77–1.41 (1.05)	205 (11.7)	3.91 (3.57, 4.29)	1.16 (1.06, 1.27)	1.17 (1.07, 1.28)	2.54 (2.30, 2.80)	1.12 (1.04, 1.20)	1.12 (1.04, 1.20)
1.41–8.22 (2.57)	207 (10.4)	4.30 (3.74, 4.96)	1.17 (1.05, 1.30)	1.12 (1.02, 1.24)	2.73 (2.44, 3.06)	1.11 (1.02, 1.21)	1.08 (1.00, 1.17)
*p*-Trend^*e*^			< 0.001	0.003		0.001	0.007
Fall/winter season
0	713 (48.9)	3.97 (3.59, 4.39)	1 (reference)	1 (reference)	2.65 (2.45, 2.87)	1 (reference)	1 (reference)
0.001–0.34 (0.10)	201 (13.6)	3.77 (3.33, 4.28)	1.04 (0.93, 1.15)	1.04 (0.94, 1.15)	2.43 (2.19, 2.70)	1.00 (0.91, 1.09)	1.01 (0.93, 1.09)
0.34–0.81 (0.59)	198 (12.6)	4.56 (4.06, 5.13)	1.22 (1.08, 1.37)	1.16 (1.04, 1.30)	2.89 (2.54, 3.29)	1.14 (1.03, 1.27)	1.09 (0.99, 1.19)
0.81–1.48 (1.09)	198 (12.8)	4.79 (4.21, 5.46)	1.13 (0.99, 1.28)	1.09 (0.97, 1.23)	3.11 (2.75, 3.52)	1.11 (0.96, 1.27)	1.07 (0.93, 1.22)
1.48–8.89 (2.69)	200 (12.3)	5.22 (4.42, 6.17)	1.17 (1.01, 1.34)	1.13 (0.99, 1.28)	3.50 (3.13, 3.92)	1.24 (1.12, 1.36)	1.20 (1.09, 1.31)
*p*-Trend^*e*^			0.003	0.010		0.002	0.009
*p*-Interaction^*f*^			0.540	0.762		0.240	0.240
Notes: CI, confidence interval; DMA, dimethylarsinate; GMR, geometric mean ratio. ^***a***^Intake values for a quantile are range (mean). ^***b***^Model 1 adjusted for urine creatinine (natural log–transformed continuous), age (continuous), sex (male/female), race/ethnicity (non-Hispanic white/non-Hispanic black/Mexican-American/Other, including multiple), education (< high school/high school or equivalent/> high school), body mass index (continuous), smoking status (never/former/current), serum cotinine (natural log–transformed continuous), poverty income ratio (PIR, continuous), and tap water source (community supply/well, cistern/spring/other/no tap water). ^***c***^Model 2 further adjusted for past 24 hr intake of rice, cereal, juice, and wine (g/kg body weight, continuous). ^***d***^All percentages are weighted to account for NHANES complex sampling design and weights. ^***e***^*p*-Trend obtained from adding quartile of poultry intake as continuous variable to model. ^***f***^*p*-Interaction obtained from Model 2, adding poultry intake as natural log–transformed continuous, season (fall, winter/spring, summer), and an interaction term for season and poultry intake.							

## Discussion

In this representative study of the U.S. population conducted when roxarsone, nitarsone, and other arsenicals were widely used in poultry production, past 24-hr consumption of poultry was associated with elevated total arsenic and DMA concentrations in urine, reflecting arsenic exposure (Chapman and Johnson 2002; Nachman et al. 2012). As hypothesized, chicken consumption was associated with increased urine total arsenic and DMA year-round, whereas only turkey consumption during the fall/winter but not during the spring/summer was associated with increased total arsenic and DMA in urine. These findings are consistent with the reported seasonal use of nitarsone in turkey production and the year-round use of roxarsone in chicken production and add to a growing body of literature suggesting that the use of arsenicals in poultry feed results in arsenic exposure for poultry consumers (Aubrey 2013).

Arsenic-based drugs were used in U.S. poultry production, which covers > 99% of the U.S. market share, for decades to prevent histomoniasis (blackhead disease) and coccidiosis (parasitic infection) and to improve weight gain and meat pigmentation (Abraham et al. 2013; Chapman and Johnson 2002; Silbergeld and Nachman 2008). Our results strongly support the decision of the FDA to withdraw approval for nitarsone sales in the United States beginning in December 2015 and for roxarsone and other arsenicals in 2013. However, there is no indication that the marketing and use of arsenicals will be discontinued internationally (FDA 2015; Yao et al. 2013).

Inorganic arsenic is an established human carcinogen that causes cancers of the lung, skin, and bladder and possibly cancers of the liver and kidney (ATSDR 2007; IARC 2012b). Increasing evidence supports that chronic low to moderate iAs exposure results in numerous noncancerous health effects, including cardiovascular, kidney, and respiratory disease; diabetes; and cognitive and reproductive defects (Ahmad et al. 2001; Chen et al. 2011; Farzan et al. 2013, 2015; Moon et al. 2012, 2013; Navas-Acien et al. 2008; Rahman et al. 2009; Tolins et al. 2014; Zheng et al. 2014). In 2011, the FDA concluded that any animal feed additive that contributed to increased iAs levels in poultry tissues was of concern (FDA 2011). The U.S. EPA’s Integrated Risk Information System (IRIS) is reevaluating iAs risk assessment at the present time; a draft appearing on the U.S. EPA website proposed an updated lung and bladder cancer potency factor of 25.7 for the U.S. population, citing the increased susceptibility of women (U.S. EPA 2010). Using this proposed cancer potency factor and intake rates from NHANES 2003–2006, Nachman et al. (2013) estimated that, assuming roxarsone use in chickens, a typical consumer of conventionally produced chicken would receive an average daily iAs dose of 1.44 × 10^–6^ mg/kg body weight, resulting in an excess 124 bladder and/or lung cancer cases per year in the United States.

Food is the primary source of unregulated arsenic exposure, highlighting the importance of eliminating or reducing dietary iAs exposures where possible (Georgopoulos et al. 2008). Specifically, rice, wine, juices, and cereals contribute to iAs exposure, and rice can also contribute to DMA exposure, whereas seafood contributes to low-toxicity organic arsenicals (Davis et al. 2012; Jackson et al. 2012; Navas-Acien et al. 2011; Schoof et al. 1999; Tariba 2011). Contamination of rice, grain, and grape products is likely attributable to the historical application of arsenic-based pesticides, naturally occurring ground water and soil contamination, and particularly for rice, the accumulation and deposition of arsenic into the rice grain (Carey et al. 2012; Chen et al. 2015; Robinson et al. 2007; Tariba 2011; Wilson et al. 2012). There are also some reports of poultry waste being used to fertilize rice paddies and roxarsone potentially contributing to iAs in the rice (*Alter v. Pfizer Inc*. 2012). Nontoxic, organic arsenicals (arsenobetaine, arsenosugars, arsenolipids) in seafood likely arise from the metabolism of naturally occurring arsenic in sea animals and plants (Sabbioni et al. 1991). Although phytoremediation by arsenic-accumulating plants can successfully remediate arsenic-contaminated crop areas, remediation may require multiple cycles over long periods of time (Hettick et al. 2015). In contrast, eliminating the unnecessary and deliberate use of arsenic-based drugs in poultry production is an easily controlled method of reducing dietary arsenic exposure.

Our seasonally stratified analysis provides strong suggestive evidence that arsenic exposure from poultry consumption was the result of arsenic-based drug use. Multiple studies have shown that roxarsone is transformed into inorganic and other arsenic species under particular environmental conditions (Arai et al. 2003; Garbarino et al. 2003; Jackson and Bertsch 2001). Elevated total and inorganic arsenic is found in poultry tissues and meat after treatment with arsenicals (Conklin et al. 2012; FDA 2011), and conventionally produced poultry is known to have higher levels of total and inorganic arsenic than organic and antibiotic-free poultry (Nachman et al. 2013). In chicken feeding experiments, a roxarsone diet resulted in elevated iAs, MMA, DMA, and roxarsone compared with a roxarsone-free diet (Liu et al. 2016). These findings are compelling, although it is always possible that other arsenic sources are responsible for elevated arsenic in poultry tissue, such as accidental or naturally occurring contamination of the soil, water, food supply, or packaging process (Hettick et al. 2015).

Our study has several limitations. We were unable to differentiate between poultry produced with and without arsenicals because information regarding the consumption of organic or antibiotic-free poultry was not available, and we were unable to quantify arsenic levels present in the consumed poultry meat. Although consumption of poultry produced without arsenic-based drugs differs across socioeconomic groups, our analysis found no differences in urine total arsenic and DMA by poultry intake across racial/ethnic groups (Figure 1), poverty–income ratio (≤ 1 vs. > 1), or education (data not shown) (Onyango et al. 2007). The USDA food code items can also be subject to substantial misclassification, in particular with regard to distinguishing between chicken and turkey intake, because many of the codes list both types of poultry together. Twenty-four–hour dietary recalls and USDA food codes can also be limited in the adjustment for potential confounding by rice, wine, and juice intake. The LODs for arsenic species in NHANES are relatively high, and ~30% of our study population had undetectable DMA concentrations. Sensitivity analyses using multiple imputed chained equations to impute DMA values below the LOD showed similar trends for turkey and chicken intake and in seasonally stratified analyses, although some associations did not reach statistical significance. In addition, neither roxarsone nor nitarsone species were analyzed in urine. Additionally, our analysis was limited to poultry consumption in the past 24 hr, and urine arsenic may reflect dietary consumption over the past 1–4 days (CDC 2013). Because urine DMA has a shorter half-life than total arsenic, urine DMA is more likely to reflect past 24-hr dietary consumption than urine total arsenic (Fowler et al. 2007). Moreover, DMA levels may also reflect individual methylation patterns of iAs to MMA and DMA, which has implications for toxicity because DMA is generally regarded as less toxic than MMA (Hughes 2002). We controlled for other dietary sources of arsenic exposure, including rice, wine, juices, cereals, and seafood. We also adjusted for sociodemographic and lifestyle changes, although we could not account for drinking water with high arsenic levels because this information was not available. Residual or uncontrolled confounding is always a possible source of bias in observational studies. Restricting to participants with undetectable arsenobetaine likely removed the contribution of nontoxic organic seafood arsenicals and their metabolites, which, at very high concentrations, can overwhelm the evaluation of other dietary sources of arsenic. Although restriction to participants with undetectable arsenobetaine levels is the most reasonable approach to remove the contribution of seafood arsenicals, some limitations exist, including the possibility of exposure misclassification. Given the current price of fishmeal, however, it is unlikely that seafood products were added as sources of protein to poultry feed. If arsenobetaine had been present in consumed poultry meat, restricting to participants with undetectable arsenobetaine could have resulted in an overcorrection. This possibility was unlikely because in our subsample with undetectable arsenobetaine, the prevalence of chicken intake was common and did not differ from that of the subsample with detectable arsenobetaine. Although restriction markedly reduced the sample size, population characteristics before and after restriction remained similar (Table 1), and our results remained robust after full adjustment for both sociodemographic and dietary factors (Table 2).

## Conclusions

Consistent with a growing body of literature establishing diet as an unregulated yet important source of arsenic exposure in the U.S. population (Jackson et al. 2012; Tariba 2011), our results indicate that the use of arsenicals in poultry production resulted in arsenic exposure to poultry consumers as measured in elevated urine total arsenic and DMA. Historical seasonal use of nitarsone in turkey production and year-round use of roxarsone in chicken production may represent an important source of chronic arsenic exposure in the U.S. population. Future research should evaluate if the relationship between poultry consumption and elevated urine total arsenic and DMA is attenuated in the years after the withdrawal of arsenic-based drugs from the U.S. market. Our study provides strong evidence to support the FDA’s recent decision to withdraw approval for nitarsone and to extend the banning of arsenic-based drugs in food production to all countries throughout the world.

## Supplemental Material

(107 KB) PDF
