# Supplementary material for: Poultry Consumption and Arsenic Exposure in the U.S. Population
Source: Environ Health Perspect. 2016 Oct 13;125(3):370–7. doi: 10.1289/EHP351 (PMC5332189; doi:10.1289/EHP351)
Supplement: (107 KB) PDF [file EHP351.s001.acco.pdf]

Environ Health Perspect
DOI: 10.1289/EHP351

Note to readers with disabilities: *EHP* strives to ensure that all journal content is accessible to all readers. However, some figures and Supplemental Material published in *EHP* articles may not conform to 508 standards due to the complexity of the information being presented. If you need assistance accessing journal content, please contact ehponline@niehs.nih.gov. Our staff will work with you to assess and meet your accessibility needs within 3 working days.

Supplemental Material

Poultry Consumption and Arsenic Exposure in the U.S. Population

Anne E. Nigra, Keeve E. Nachman, David C. Love, Maria Grau-Perez, and Ana Navas-Acien

Table of Contents

Table S1. Food Commodity Index Database (FCID) commodity codes used to define intake variables.
Table S2. USDA food codes used to define cereal intake.

Table S1. Food Commodity Index Database (FCID) commodity codes used to define intake variables.

Intake variable	FCID commodity codes	Description
Poultry	4000093000	Chicken, meat
	5000382000	Turkey, meat
Chicken	4000093000	Chicken, meat
Turkey	5000382000	Turkey, meat
Rice	1500323000	Rice, white
	1500324000	Rice, brown
	1500325000	Rice, bran
	1500326000	Rice, flour
Wine	1304179000	Grape, wine and sherry
Juice	1100010000	Apple, juice
	1304176000	Grape, juice
	1100268000	Pear, juice
	1203288000	Prune, juice
	1307132000	Cranberry, juice

Table S2. USDA food codes used to define cereal intake.

Intake variable	USDA food code	Description
Cereal	56200300 through 56203620	Pastas, cooked cereals, and rice
	56206970 through 57604100	Cereals, not specified as to cooked, or not cooked, ready-to-eat cereals, and cereal grains
